# Validation of a French version of the Freiburg Mindfulness Inventory - short version: relationships between mindfulness and stress in an adult population

**DOI:** 10.1186/1751-0759-4-8

**Published:** 2010-08-12

**Authors:** Marion Trousselard, Dominique Steiler, Christian Raphel, Corinne Cian, Raffi Duymedjian, Damien Claverie, Frédéric Canini

**Affiliations:** 1 Département des Facteurs Humains, Centre de recherches du service de santé des Armées, 24 avenue des maquis du Grésivaudan, BP 87, 38702 La Tronche cedex, France; 2 Département Management et Comportements, Grenoble Ecole de Management, 12 rue Pierre Sémard, BP 127, 38003 Grenoble cedex 01, France

## Abstract

**Background:**

Whereas interest in incorporating mindfulness into interventions in medicine is growing, data on the relationships of mindfulness to stress and coping in management is still scarce. This report first presents a French validation of the Freiburg Mindfulness Inventory-short form (FMI) in a middle-aged working population. Secondly, it investigates the relationship between psychological adjustment and mindfulness.

**Methods:**

Five hundred and six non-clinical middle-aged working individuals rated themselves on the self-report French version FMI and completed measures of psychological constructs potentially related to mindfulness levels.

**Results:**

Results were comparable to results of the original short version. Internal consistency of the scale based on the one-factor solution was .74, and test-retest reliability was good. The one-dimensional solution as the alternative to the two-factor structure solution yielded suboptimal fit indices. Correlations also indicated that individuals scoring high on mindfulness are prone to stress tolerance, positive affects and higher self-efficacy. Furthermore, subjects with no reports of stressful events were higher on mindfulness.

**Conclusion:**

These data showed that mindfulness can be measured validly and reliably with the proposed French version of the FMI. The data also highlighted the relationship between mindfulness and stress in an adult population. Mindfulness appears to reduce negative appraisals of challenging or threatening events.

## Background

Mindfulness has been described as a non-elaborative, non-judgmental present-centred awareness in which each thought, feeling or sensation that arises in the attention field is acknowledged and accepted as it is [1-4]. Mindfulness appears as an attribute of consciousness long believed to promote well-being [5,6]. Indeed, mindfulness training is related to positive psychological and physiological outcomes [6,7]. A high level of mindfulness increases willingness to tolerate uncomfortable emotions and sensations [8-11] and emotional acceptance [12,13,4]. It also decreases the impact of negative emotional events and reduces time needed to recover [12]. Mindfulness is therefore employed in the treatment of various anxiety disorders, for example in the non-clinical population for helping to cope with challenging or threatening events [12,14-17]. Thus, it can serve as a predictor of day-to-day self-regulated behaviour and adaptability to stressful events.

Over the past 10 years, several instruments have been developed to measure dispositional mindfulness. Six main scales are available: the Mindful Attention Awareness Scale (MAAS; 10), the Kentucky Inventory of Mindfulness Skills (KIMS; 18), the Cognitive and Affective Mindfulness Scale (CAMS; 12), the Toronto Mindfulness Scale (TMS; 17), the Mindfulness Questionnaire (MQ; Chadwick P, Hember M, Mead S, Lilley B, Dagnan D: Responding mindfully to unpleasant thoughts and images: reliability and validity of the Mindfulness Questionnaire, submitted), and the Freiburg Mindfulness Inventory (FMI; 7). Despite the fact that all of these tools use self-reported assessment methods to explore mindfulness and that correlations between them were found, differences do separate them [18]. More specifically, MAAS is a 15-item instrument only focusing on attention and awareness without assessing acceptance. KIMS, developed from Linehan's Dialectical Behavior Therapy, is a 39-item tool used mainly for therapy effectiveness. CAMS is a 12-item inventory designed to measure attention, awareness, present-focus and acceptance in general daily occurrences. TMS is a 13-item instrument uniquely state oriented and is used for meditation research. MQ investigates mindfulness of the distressing thoughts and images of the mind by using a 16-item instrument. FMI has been developed qualitatively out of the original Buddhist concept of mindfulness. In its long form (30 items), it measures mindfulness as a general construct that has some interrelated attention, awareness and acceptance facets. However, it is difficult to apply to people without any background knowledge of mindfulness. The published short form (14 items) captures all aspects of the long form [7,19]. It is semantically independent from a Buddhist or meditation context and is applicable to all population groups.

Whether mindfulness can be looked at from different angles, to capture the nature of the concept implies analyses of all questionnaires in parallel [7,18]. On one hand, although most mindfulness measures are one-dimensional and self-reported, the multidimensional nature of mindfulness has been taken into account by several authors [18]. Baer et al. (2006), combining several mindfulness scales (MAAS, KIMS, FMI, CAMS, MQ) into a single data set of 112 items, found five identifiable factors that are internally consistent and only modestly correlated with each other [20]. The identifiable factors were: Observing, Describing, Acting with awareness, Nonjudging and Nonreactivity to inner experience. Derived from these studies and based on skills as defined in the Dialectical Behavior Therapy, these authors developed the 39 item version of the Five Facets Mindfulness Questionnaire (FFMQ). Further, Kohls et al. (2009) tested a one-dimensional and an alternative two-dimensional solution (Presence and Acceptance) of the FMI-14 [19]. Whereas results suggested a heuristic value in the two-factorial solution, the one dimensional approach appeared sufficient for practical purposes. On the other hand, to what extent it is related to other constructs known to be predictive of psychological symptoms is a matter for further elucidation and investigation. For example, using KIMS and MAAS, the authors found that higher scores of mindfulness were associated with higher body satisfaction (Body Cathexis Scale; [21]), less social anxiety (Scale for Interpersonal Behaviour; [22]). With the KIMS, it has been shown that subjects scoring high in mindfulness exhibited better identification and description of feeling (Toronto Alexithymia Scale-20; [23,24]). Further, mindfulness scores on the TMS were correlated with measures of the situational self-awareness (Situational Self-Awareness Scale; [25]). For the FMI-14, a negative correlation was observed with anxiety and depression which is entirely due to the Acceptance factor of mindfulness [19]. Thus, the exploration of the relationship between the existing scales of mindfulness and several measures of psychological constructs showed positive correlations with positive personality trait and well-being indicators and negative correlations with neuroticism and emotional disturbance measures [5,7,18]. Baer et al. (2006) have specified which facets of mindfulness were responsible for these correlates [20]. They also showed that meditation experience influenced the relationship between facets of mindfulness (FFMQ) and psychological scales [26].

Finally, the uni-/multi-dimensional nature of mindfulness and its relation to other variables require further investigations involving different languages and cultures. Whereas interest in incorporating mindfulness into interventions in medicine and stress and coping in management has been increasing in recent times, French professionals coming to this field have no French language scale to assess mindfulness, its dimensional nature or its effectiveness for coping with stress.

### Aims

The current study first aims to present a French validation of the short form of the FMI. The justification for choice of the FMI short form is two-fold: this scale has both a broader application in clinical contexts and in differential research contexts involving non-clinical individuals [7,18,19,26]. The psychometric properties of the French FMI version were investigated in a non-clinical middle-aged working group. As the majority of the previous studies were conducted using students, little is known about how mindfulness operates in a general working population. The relationships between the tested French FMI version and psychological dimensions known to be predictors of psychological well-being and emotional disturbances are assessed. Confronted with the role mindfulness plays on emotional well-being (observed with English self-rating instruments [5,7,18,27]), it is expected that subjects scoring higher in the French FMI version would score higher on the indicators of well-being and would score lower in the indicators of psychological disturbances. The second goal of the present study is to evaluate the relationship between mindfulness and stress in an adult population.

## Method

### Procedure and participants

The French translation process of the FMI was completed in two steps. Firstly, a committee of four collaborators who were fluent in both French and English revised a first translation of the scale, resolving translation difficulties by consensus. Secondly, a back-translation procedure was used. The initial translation (English to French) was followed by the back-translation (French to English) done by three bilingual English native speakers without using the original version. This translation/back-translation process was repeated twice, with committee evaluation and recommendations being made between the two translation/back-translation processes. At each stage, two external experts in the field of psychological assessments (French and English experts) were asked to examine the translation of each specific item.

The final French version of the FMI was included in a set of self-report questionnaires composed of two parts. The first part included questionnaires assessing common socio-demographic data and FMI. The second part was composed of three useful psychological questionnaires for the study of promoting stress adaptability. Companies from the haulage, information technology (IT), and automotive sectors were contacted for the study. One company from each sector accepted to participate. For each company, individuals received a cover letter supported by their respective board to invite them to participate in the study. The cover letter contained three types of information. Firstly, the main aim of the study was noted as a validation of the French translation of a psychological questionnaire with guidance for completion of the instruments. Secondly, there were two criteria to be included in the study: (*i*) not to be undergoing treatment and (*ii*) not to have a personal interest in mindfulness. The set of questionnaires were presented online on each company's intranet portal from January to September 2008 (Time 1: Baseline). To ensure data quality, the guidelines for internet-based experimenting as presented by Reips (2002) were followed [28]. Each answer was coded to ensure confidentiality and the possibility of monitoring. Anonymous volunteers completed assessment measures online in the intranet portal of their respective companies in a single session and submitted their answers. Responses were excluded from data collection if the first part of the set of questionnaires (socio-demographic data and FMI) was not fully completed. In total, five hundred and six working individuals (236 females and 270 males) participated in the study by completing at least the first part of the questionnaires (biographical data and FMI). Most of them completed two of the three questionnaires of the second part. Only 53 participants (around 10%) completed the whole set of questionnaires. The study was conducted in accordance with all applicable regulatory requirements, including the 1996 version of the Declaration of Helsinki and approved by the ethics committee of the French Military Health Service. All volunteers gave written informed consent before participation.

In July 2008 (six months after the initiation of the questionnaire), the companies were once more contacted for test-retest reliability. Only the private transportation and logistics companies participated. By using a second cover letter, participants were invited to complete again on their online portals the common biographical data and the FMI. The cover letter contained two types of information. Firstly, the aim was noted as being to establish test-retest reliability of the French translation of the psychological questionnaire. Secondly, three criteria were necessary: (*i*) to have completed the first part in January or February 2008 (checked through the computerized code), *(ii) *not to be undergoing therapeutic treatment and (*iii*) not having a personal interest in mindfulness. Access to the set of questionnaires was closed in September 2008 (Time 2). Only fifty-three individuals completed the FMI. Data from individuals were matched according to the confidentiality code.

### Measures

The socio-demographic data included age, ethnicity, gender, educational level, job's features, and matrimonial situation. Subjects were also questioned about the presence (response yes) or not (response no) of stressful events in the last two years. The only instruction given for that purpose was to answer "yes" or "no" for both their private and professional life.

The Freiburg Mindfulness Inventory-14 is a short form with 14 items developed for people without any background knowledge in mindfulness [7]. It constitutes a consistent and reliable scale evaluating several important aspects of mindfulness, which is considered as one-dimensional for practical purposes [7,19]. Each self-descriptive statement was evaluated using a four-point Likert scale ranging from 1 (strongly disagree) to 4 (strongly agree). Depending on the suggested time frame, state-and trait-like components could be assessed. In the present study, the short form was used for measuring mindfulness-trait (Additional file 1).

The Perceived Stress Scale (PSS; [29-31]) is a 14-item scale designed to assess subjects' appraisal of how stressful their life situation feels to them. The PSS is recommended for assessing non-specific appraisal because it is found to predict better stress-related psychological symptoms and physical symptoms compared to commonly used life event scales [32,33]. Because stress-tolerant individuals have lower perceived stress scores than those lacking stress-tolerance skills [33], a negative correlation was expected with FMI scores.

The general Self-Efficacy Scale consists of a 17-item self report measure that asks the subjects to rate their confidence in their ability to be consistently successful in organising and implementing the courses of action required to produce given accomplishments [34,35]. One measure of general self-efficacy was obtained which was specifically designed for managers [36]. General self-efficacy is found to be an important aspect of functioning in a variety of realms [34,37]. The beneficial effects of self-efficacy include coping with trauma [34] and performance [34]. Since mindfulness includes awareness and acceptance of all experiences and actions, a positive correlation between the Self-Efficacy Scale scores and the FMI scores was expected.

The Positive and Negative Affect Schedule consists of two scales that assess positive and negative affect, respectively (PANAS; [29,38,39]). Each scale has a ten-word emotion descriptor and respondent rating conveying how well each descriptor reflects their current emotions. Each word was evaluated using a scale of one to five, as to whether the word fits the usual or time-limited state of the individual. In this study, the general or usual state was requested. Negative correlations with PANAS negative affects and positive correlations with PANAS positive affects were observed for the MAAS [5]. Similar correlates were predicted.

### Statistical analysis

Whenever possible, parameters were expressed as mean and standard deviation (SD). All statistics were performed using the SPSS 17.0 software package, except for the factorial structure analysis, which was performed with the AMOS 18.0 software package (SPSS Inc., Chicago, IL, USA). Inter-sample differences were studied using Student's "t"-test or chi-square. The validation of the FMI translation was assessed in three steps. Firstly, the sensitivity and reliability of the French version of the questionnaire were examined. The inter-individual sensitivity was evaluated using the normality of the distribution of the participants' FMI scores. A second index of inter-individual sensitivity was the degree to which scores on the scale discriminated members of the group. Reliability was performed using Cronbach's Alpha, as well as the intra-class correlation coefficients (test-retest fidelity). Secondly, the factorial structure was investigated in accordance with the procedure used for the original FMI version [7,19]. Due to the ambiguity in factorial structure of the original version [40], the two factorial proposed solutions were considered. Finally, construct validity was also assessed by analyzing the correlations between the FMI scores and the scores for measures of psychological variables using Pearson correlation coefficients.

## Results

### Socio-demographic Sample

The descriptive data showed that 53.16% of the participants were men, 61.6% were aged between 21 and 36 years, more than 80% were white, 66.99% were married or living as couples, and 58.7% completed undergraduate educational level (Table 1). Most of them worked in a large company (automotive or IT companies) and the remaining in a small one (haulage company). Three-quarters of the participants reported experience of a recent stressful event in last year. As no significant difference was observed for school education level (t-test, p > .05), for age, ethnicity, matrimonial situation, or for reports of stressful events (chi-square, p > .05) between subjects according to the company, data from all companies were grouped together for further analyses.

**Table 1:** Scores (Standard Deviations) of the French FMI version for the non-clinical middle-aged sample according to the age, gender, meditation experience, and stressful event reported.

Non-clinical middle-aged sample (506)						
**Variables**		**n**	**%**	**FMI**	**t values**	**p**

Age (group)	< 36 years	316	61.6	38.5 (5.1)	t = -2.56	p < .01
						
	> 37 years	190	37.4	39.7 (5.5)		

Gender	Men	269	53.16	39.24 (5.35)	t = 1.21	p > .05
	Women	235	46.84	38.68 (5.57)		

Marital status	Married or as couple	339	66.99	39.3(5.38)	t = 1.28	p > .05
	Divorced	66	13.04	38.67(5.52)		
	Single	101	19.96			

Educational level	Undergraduate studies	210	41.3	38.71 (5.75)	t = 1.04	p > .05
	graduate studies	296	58.7	39.21 (5.12)		

Employment status	Middle managers	405	80.04	38.87(5.3)	t = 1.35	p > .05
	Top managers	101	19.96	39.1(5.5)		

Companies	Small		34.6	38.72(5.34)	t = 1.52	p > .05
	Large		65.4	39.1(5.46)		

Relaxation experience	No	473	93.48	38.9 (5.18)	t = 1.75	p = .08
	Yes	33	6.52	40.62 (6.04)		

Stressful event reported	No	132	26.09	40.5 (4.8)	t = 3.83	p < .001
	Yes	367	72.53	38.5 (5.5)		
	No response	7	1.38	40.46		

Thirty-three participants only reported having experience in relaxation techniques (n = 11), yoga (n = 12) or martial arts (n = 10) and only thirteen of them were actually practising. The length of time of practice was 3.2 years on average (SD = 3.56). They were not different from subjects without such experience for school education level, age, ethnicity, matrimonial situation, or for reports of stressful events between (chi-square, p > .05).

### Internal validity

The internal validity (consistency) of the French FMI version could be considered as acceptable if this tool is consistent and accurate. Results (Table 2) obtained in the original short FMI version were almost entirely reproduced in the French version when correlating every item of the instrument with the others using Cronbach's alpha coefficient of internal consistency (α; [41]). However, when considering separately the correlation between items and scales in each group, item 13 (e.g. "I'm impatient with myself and with others") did not correlate well (r_it13 _< .20). As item 13 did not appear to contribute significantly to internal consistency, "alpha if item 13 deleted" was calculated (Table 2). When item 13 was deleted, the psychometric properties of French FMI-13 were improved (Table 2). The temporal stability of the scale over a period of 6 months was examined in a sub sample of the participants (N = 53). This sub-sample did not differ from the full population at baseline (Time 1) for FMI score, school education level, age, ethnicity, stressful event reported or for matrimonial situation (chi-square, p > .05). The intra-class correlation (ICC) coefficient (absolute agreement coefficient) using a two-factor model of ICC was applied to the data collected in the sub-sample for test-retest reliability at six months. The ICC coefficient was .80 (p < .01), indicating a high reliability for this French version.

**Table 2:** Statistical properties of the French FMI version (14-item and 13-item version) for the non-clinical middle-aged sample in comparison to the original data [7].

Sample (n)	Original version (74)	French version (506)	
Form	14 items	14 items	Item 13 deleted

Mean	37.24	38.98	36.08

SD	5.63	5.43	5.45

Range (theoretical)	25-52 (14-56)	14-56 (14-56)	13-52

Kurtosis	.08	1.12	.97

Skewness	-.4	-.28	-.26

Cronbach's α	.79	.74	.77

Mean item-inter-correlation	.21	.17	.21

### Construct validity

The French FMI version can be considered as a valid instrument if it is effective in measuring the attribute that it is theoretically supposed to measure. The absence of an alternative French instrument for measuring mindfulness prevented assessing the degree to which the construct itself is actually measured. Thus, the construct validity-related data were gathered using two methods.

Firstly, for face and content validity, during back-translation, non-psychometric judgment from ten non-research respondents, the committee itself and scientific experts were questioned on the apparent quality of the items. Less than 5% of the subjects judged the questionnaire of little interest to them. As this study focused on the trans-cultural validation of a measuring tool, this validity procedure was important in order to be sure that the translation transcribed the original version items accurately.

Secondly, the structural framework of the items was studied. In a first step, an exploratory principal component factor analysis oblique rotation was applied, as previous studies found correlated factors for the FMI [7,19]. Results showed that the Kaiser-Meyer-Oklin measure of sampling adequacy (.82) and the Bartlett test of sphericity statistic (1112.5, [91df], p < .001) were suitable for the factor analysis. The number of factors was determined by the Scree Test and the interpretability of these factors. These criteria suggested a four-factor solution with 51.46% explanation of variance (eigenvalues: 3.51, 1.51, 1.1, and 1.07). Tabachnick and Fidell (2001) suggest that, in exploratory factor analysis, one item forms one part of a factor if its factor loading on that specific factor is at least .32 and at least .1 greater than its other factor loadings [42]. However, the translated items were not readily separable since half of the items (items 2, 5, 7, 8, 10, 11 and 12) did not meet these criteria. Moreover, the remaining items (items 1, 3, 4, 6, 9, 13, 14) did not duplicate one of the depicted sub-factors (Acceptance or Presence) in the two-factor alternative solution.

**Figure 1:**
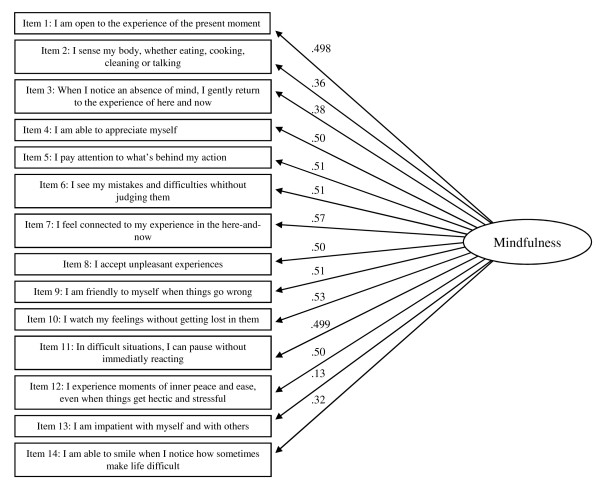
**Confirmatory factor analysis for FMI (full scale-14 items), unidimensional solution (N = 506)**. Note: rectangles indicate observed indicator variables for the FMI. The oval indicates the construct mindfulness as unobserved latent variable. Numbers printed bold at single-headed arrows indicate standardized regression weights.

**Figure 2:**
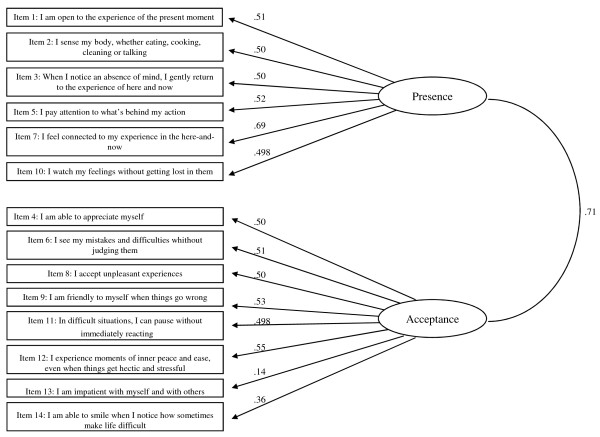
**Confirmatory factor analysis for FMI (full scale-14 items) - two-unidimensional solution (N = 506) suggested by Kohls et al**. (2009; 19).

In a second step, two Confirmatory Factorial Analyses (CFA) using maximum likelihood were undertaken on item responses from the population sample to test the appropriateness of the Structural Equation Models (SEM): one for the one-factor solution structure (Figure 1) and an alternative for the two-factor structure solution (Figure 2). To assess fit, it is generally recognized that it is advantageous to use several indexes per construct [30]. Four measures were used to assess fit in the present study: chi-square/degree of freedom ratio (CMINI/*df*), Goodness of Fit Index (GFI), Adjusted Goodness of Fit Index (AGFI) and Root Mean Square of Approximation (RMSEA), with their desired levels being < 3, > 0.9, > .85 and <.06 respectively. Both the one and two-factor solutions yielded good fit indices for GFI (.92 for both the one and the two-factor models) and AGFI (.90 and .89 respectively for the one factor model and the two-factor model). But, indices for CMINI/*df *(.4.1 and 3.55 respectively for the one factor model and the two-factor model) and RMSEA (.07 for both the one and two-factor models) were slightly above the limit suggested [37]. All items loaded > .05 onto the single factor for the one factor solution except items 2, 3, 13. For the two-factor solution, all items loaded > .05, except item 13, and 14 (sub-factor Presence).

### Criterion validity

Criterion validity means discriminative accuracy, or ability to acknowledge differences between participants. The ideal distribution usually expected is a "normal distribution". Means, standard deviation, Skewness and Kurtosis are used to describe the distribution and to observe sensitivity (Table 2). The normality assumption investigated using the Shapiro-Wilk test on the sample showed significant non-Normality (p < .01). The kurtosis was markedly larger than the original version meaning a distribution with more extreme responses.

Results of inter-individual sensitivity are given in Table 1. No gender difference on FMI scores was observed. The FMI score appeared independent of the educational level, of the company, as of the matrimonial status. There was a significant effect observed due to age in the population with the FMI score highest in the age group between 37 and 55 years. Furthermore, the FMI score appeared associated with relaxation, yoga or martial arts experience. When regarding the reported experience of a recent stressful event on FMI scores, results showed that subjects who did not report such an event scored higher on FMI than subjects reporting a stressful event.

Finally, relationships with other constructs were evaluated. As 10% of participants completed all the questionnaires, correlations were separately applied considering each of the three following sub-samples of respondents: the 200 respondents who answered both PANAS and FMI, the 206 of respondents who answered both general Self-Efficacy Scale and FMI, and the 209 respondents who answered both PSS and FMI. In the considered samples, means (SD) were 22.59 (6.71) for PANAS (negative affect), 37.45 (4.18) for PANAS (positive affect), 54.58 (6.52) for Self-Efficacy and 34.35 (6.11) for PSS scales. Correlates between the one dimensional French FMI solution and other constructs are given in table 3. These correlations remained significant after controlling for the report of an experienced stressful event (Table 3).

**Table 3:** Pearson's correlation coefficients between FMI (one-dimensional), FMI (two-dimensional: subscales Presence and Acceptance) and the selected psychological constructs according to the sub-samples of responders.

	*Predicted negative correlations*		*Predicted positive correlations*	
**Scales and subscales**	**PSS (n = 209)**	**PANAS-Negative** **affect (n = 200)**	**PANAS-Positive** **affect (n = 200)**	**Self-Efficacy (n = 206)**

**FMI-Mindfulness**	-.56*	-.32*	.53*	.43*

Controlled by SE^(1)^	-.54*	-.28*	.32*	.42*

**FMI-Presence (6 items)**	-.47*	-.22*	.48*	.42*

Controlled by SE^(1)^	-.46*	-.22*	.38*	.42*

Controlled by the sub-factor Acceptance^(1)^	-.34**	-.32**	.31**	-.05

**FMI-Acceptance (8 items)**	-.46*	-.32*	.46*	.34*

Controlled by SE^(1)^	-.46*	-.30*	.38*	.33*

Controlled by the sub-factor Presence^(1)^	-.32**	-.37**	-.25**	.17*

* p < .01, ** p < .001

^(1)^: Pearson's partial correlation coefficients between mindfulness scale and subscales and the selected psychological constructs controlled by self-report of Stressful Events (SE). Partial correlations between FMI subscales (either Presence or Acceptance) and the selected psychological constructs with controlling for the respective other FMI subscale.

In order to test the two-factor alternative construct, correlation analyses were first applied between the one-dimensional and the two dimensional (Presence and Acceptance) solution for the total population. Results showed positive correlations between the FMI one-dimensional solution and the FMI sub-factor Presence and Acceptance (r = .81, p < .001 and r = .89, p < .001, respectively). The sub-factor Presence was positively correlated to the sub-factor Acceptance (r = .48, p < .001). In a second step, correlations were applied between the FMI sub-factor Presence and Acceptance and PANAS, Self-Efficacy scale, and PSS for the three sub-samples, separately (Table 3). These correlations remained significant after controlling for the report of an experienced stressful event. In a third step, partial correlations of the FMI, controlling either Presence or Acceptance, were independently computed for each sub-sample separately. Partial correlations indicated that after controlling for Presence or Acceptance, the significant correlations were shown to remain significant, except between the FMI sub-factor Presence and the PANAS-negative affect after controlling for Acceptance (Table 3).

## Discussion

This study assessed the psychometric properties of a French translation of the 14-item FMI in a sample of non-clinical middle-aged individuals. Firstly, for internal consistency, the lowest alpha coefficient was .74, and closely comparable to results of the original short version. The inter-individual sensitivity showed an acceptable normal distribution of FMI scores. The French FMI version thus appears to be a strong validation of the original FMI short version with a good temporal stability over a period of 6 months. However, item 13 did not appear to contribute significantly to internal consistency. This item concerns the ability to confront impatience. It may be considered as an outcome of mindfulness rather than the core of the mindfulness construct [20]. Baer et al. (2006) suggested that confounding elements of mindfulness with its outcome could impair the ability of a self-rating instrument to capture the nature of mindfulness [20]. It is unclear whether this item must be deleted or better translated as the original formulation was translated by several bilingual language experts.

Secondly, the one-factor solution and the alternative two-factor structure solution yielded suboptimal fit indices. However, there is ambiguity concerning the factorial structure. On one hand, as observed by Walach et al. (2006), the exploratory factorial structure showed that the translated items are not readily separable suggesting that the French translation of the FMI explores mindfulness as "a general construct that has some inter-related facets" (Walach et al., 2006; p. 1548; 7). Regarding correlation analyses on the other hand, the two-factor solution did not differ from the one-factor solution, except for the correlation between the FMI sub-factor Presence and the PANAS-negative affect after controlling for Acceptance. Using anxiety and depression scales for investigating the factorial structure, Kohls et al. (2009) observed differences between the one-factor and the two-factor solutions [19]. Consequently, they proposed to use the FMI with the one dimensional construct when mindfulness assessment is a global moderator or indicator variable and the two-dimensional alternative when focusing upon potential causal mechanisms [19]. It has been proposed that the presence of items using acceptance-related terms could account for the difficulty in defining the dimensional nature of the mindfulness [7,19,20,43]. Indeed, acceptance could mean either approval of undesirable conditions or passive resignation [7,20]. Thus, it can be speculated that the presence of five items using acceptance-related terms (items 4, 8, 9, 11, 14) from the 14 items pool of the original and French FMI could account for the difficulty for a common factorial structure. Furthermore, for Grossman (2008; p405) "serious conceptual difficulties and differences, even among experts, in a common understanding of just what mindfulness is" could explain the ambiguity concerning the factorial structure [43]. For example, from Brown and Ryan's (2004) perspective, the facet of Acceptance can be subsumed with an individual's ability of being present. Conversely, from Kohls et al. (2009) perspective, the facets of Acceptance and Presence should not be intermingled as their relations with anxiety and depression were different [19]. Finally, whether mindfulness must be considered as a multifaceted construct, or not, needs further investigation [44].

Thirdly, FMI scores appeared to depend on demographic features. FMI scores were higher for the older non-clinical population but this effect should be taken with caution because differences for age although significant are small. Namely, the question of the changes in mindfulness scores over a lifetime would merit a more precise correlation analysis using continuous variables for age instead of age bands, as it was used in this study. Furthermore, subjects with some experience in relaxation, yoga or martial arts, exhibited a tendency for a higher mindfulness level, as suggested in the literature [7,26]. Results also highlighted that FMI scores were higher for subjects without report of the experience a stressful event. This finding suggests that mindfulness may constitute an experiential mode of processing with implications for the perception of and response to stress situations [16].

When regarding the correlations between FMI scores and scores for measures of psychological variables in accordance with the expected direction, results were satisfactory. The choice of the psychological questionnaires was constrained by two main considerations: firstly, the existing French version of the measurement tools must have demonstrated good psychometric properties. Secondly, they must be pertinent for studying the link between mindfulness and some facets of stress adaptability. The PSS, and the PANAS negative emotions' instruments, which assess emotional disturbances, correlated negatively with mindfulness scores. The Self-efficacy, and the PANAS positive emotions, considered as subjective well-being measures, were positively linked with mindfulness scores. These data suggest that individuals with higher mindfulness scores may be more stress-tolerant (PSS; 33), have positive emotions (PANAS) rather than negative, a greater sense of self-efficacy, and may be somewhat less prone to report stressful events. Thus, mindfulness appears to reduce negative appraisals of challenging or threatening events [16]. Stress appraisals concern the cognitive processes through which an individual evaluates events. As mindful individuals orient themselves to ongoing events and experiences in a receptive, attentive manner, it could be suggested that a mindful disposition alters the stress process by attenuating negative appraisals of stress in demanding situations. Whether a mindful disposition could protect when faced with a traumatic event (Acute Stress Disorder) or chronic professional stress (burnout) merits further investigation.

Finally, the French translation of the FMI short version has proven to be a satisfactory measure of mindfulness, which could be proposed to a French professional coming to the field of mindfulness. One of the merits of the present study was to assess a non-clinical middle-aged population as the majority of the research has been conducted with students or clinical respondents. The French FMI version, however, needs additional studies to assess whether or not it would be sensitive to change. Investigations need to be carried out on clinical as well as on meditative samples. Another problem concerns the translation of the word "mindfulness" in the French language. The usual accepted translation is "pleine conscience". The term "conscience", however, is an ambiguous term for French individuals as it mainly focuses on cognitive processes. The question of a French title of the FMI is not resolved as the proposal to entitle it "Inventaire de Pleine Conscience de Freibourg" has not yet found consensus. Finally, the study highlights that research to establish validity for a novel trans-cultural instrument should be considered as an ongoing research process.

## Conclusion

This investigation is a psychometric analysis of a French version of the Freiburg Mindfulness Inventory in a non-clinical population. Using data from a large French working middle-aged population, the results first support the validation of the French version. Second, by investigating the relationship between mindfulness and stress appraisal for workers, we highlight the interest in incorporating mindfulness into coping with stress in management.

## Competing interests

The authors declare that they have no competing interests.

## Authors' contributions

MT and DS participated in the study design, analysis and interpretation of data and revision of the manuscript. CR and CC participated in the study design, analysis and interpretation of data and drafting the manuscript. RD participated in the translation of the French version and the revision of the manuscript. DC and FC participated in the acquisition of data and made substantive contributions to the study design, conduct, and interpretation of the results.

All authors read and approved the final manuscript.

## Supplementary Material

Additional file 1 **French translation of the FMI: « Inventaire de Pleine Conscience de Freiburg »**. The six items loading onto the sub-factor « Presence » are indicated as "P"; The height items loading onto the sub-factor « Acceptance » are indicated as "A". Click here for file
