# Supplementary material for: Validation of a French version of the Freiburg Mindfulness Inventory - short version: relationships between mindfulness and stress in an adult population
Source: Biopsychosoc Med. 2010 Aug 12;4:8. doi: 10.1186/1751-0759-4-8 (PMC2927476; doi:10.1186/1751-0759-4-8)
Supplement: Additional file 1 — French translation of the FMI: « Inventaire de Pleine Conscience de Freiburg ». The six items loading onto the sub-factor « Presence » are indicated as "P"; The height items loading onto the sub-factor « Acceptance » are indicated as "A". [file 1751-0759-4-8-S1.DOC]

**Additional file**

Additional file 1 : French translation of the FMI : « Inventaire de Pleine Conscience de Freiburg ». The six items loading onto the sub-factor « Presence » are indicated as “P”; The height items loading onto the sub-factor « Acceptance » are indicated as “A”.

Consignes : Le but de ce test est de décrire comment vous avez vécu la période des … derniers mois qui vient de s’écouler. Répondez de votre mieux à chaque déclaration. S.V.P., répondez aussi franchement et spontanément que possible. Il n’y a pas réponses « justes » ou « fausses », « bonnes » ou « mauvaises ». Ce qui est important est votre expérience personnelle.

|  |  | **Presque jamais** | **Occasion-nellement** | **Assez souvent** | **Presque toujours** |
| --- | --- | --- | --- | --- | --- |
| P | 1. Je suis réceptif (ve) à ce que je vis dans le moment présent |  |  |  |  |
| P | 2. Je ressens mon corps dans les actes de la vie quotidienne (manger, cuisiner, parler, faire le ménage,…) |  |  |  |  |
| P | 3. Lorsque je réalise que je m’égare dans mes pensées, je reviens naturellement à ce que je suis en train de vivre dans le moment présent |  |  |  |  |
| A | 4. Je suis capable d’apprécier à sa juste valeur la personne que je suis |  |  |  |  |
| P | 5. Je prête attention à ce qui me fait agir dans mon quotidien |  |  |  |  |
| A | 6. Je regarde mes erreurs et difficultés sans les juger |  |  |  |  |
| P | 7. Je suis pleinement en lien avec ce que je vis dans le moment présent |  |  |  |  |
| A | 8. J’accepte les expériences désagréables |  |  |  |  |
| A | 9. Je prends soin de moi-même lorsque les choses vont mal |  |  |  |  |
| A | 10. Je suis à l’écoute de mes sentiments sans me laisser déborder par eux |  |  |  |  |
| A | 11. Dans les situations difficiles, je sais marquer une pause avant de réagir |  |  |  |  |
| A | 12. Je vis des moments de calme et de paix intérieurs, même lorsque les choses sont mouvementées et stressantes |  |  |  |  |
| A | 13. Je suis impatient(e) envers moi-même et envers les autres |  |  |  |  |
| A | 14. Je sais sourire lorsque je me rends compte à quel point je peux parfois me rendre la vie difficile |  |  |  |  |
